# 

**Published:** 1869-04

**Authors:** 


WE are in receipt of a work from the publishing house of HENRY C. LEA, of Philadelphia, entitled a *Conspectus of the Medical Sciences*, comprising Manuals of Anatomy, Physiology, Chemistry, Materia Medica, Practice of Medicine, Surgery and Obstetrics; for the use of students: by Henry Hartshorn, A.M., M.D., Professor of Hygiene in the University of Pennsylvania, Auxiliary Faculty of Medicine, &c. The work contains over one thousand pages, with three hundred and ten illustrations. The type, paper and mechanical execution are worthy the publisher, and we have no doubt but that the *Conspectus* will meet with favor from medical students.

In our reviews and notices of books in the last issue of the JOURNAL, in reference to the *Theoretical and Practical Treatise on Midwifery*, by P. CAZEAU, of Paris, we should have said that the work contained over eleven hundred pages instead of seven hundred, and that the work of GRAILY HEWITT, M. D., F. R. C. P., on the *Diseases of Women*, contained seven huudred, instead of one hundred, as printed. These with many other new and valuable works, as presented by catalogue just received, are furnished to the profession by that well known publishing house of *Lindsay & Blackiston*, No. 25 South Sixth street, Philadelphia.

